# Are you sometimes lost? Read our new Concept Articles!

**DOI:** 10.1007/s00424-024-02963-8

**Published:** 2024-04-16

**Authors:** Andreas Draguhn, Armin Kurtz

**Affiliations:** 1 Institute for Physiology and Pathophysiology, Medical Faculty, Heidelberg University, Im Neuenheimer Feld 326, 69120 Heidelberg, Germany; 2 Institute for Physiology, University of Regensburg, Universitätsstraße 31, 93053 Regensburg, Germany

A hallmark of physiology is its systemic point of view. Most of us aim at contextualizing our specific findings within the functions of the whole organism, or even the organism and its environment. How does a particular transport mechanism affect ion homeostasis? How does a specific neuronal network oscillation support cognition? How does a newly identified gene mutation explain the symptoms of a related disease? Such questions require broad knowledge across system levels and physiological functions. This holistic view is at the heart of physiology, and it is essential for our teaching, be it in preclinical medical physiology, pharmacology, or other areas of biomedical sciences.

On the other hand, we all know that the age of scientific generalists has passed. The polarity between increasing sub-specialization and a holistic perspective is a constant challenge for every physiologist, particularly when an interdisciplinary perspective is required, as in complex systemic diseases or in the increasingly important field of body-environment interactions. How can we deal with these developments and secure a minimal level of mutual understanding across highly specialized sub-communities? As scientists, academic teachers, and journal editors, we have a canonical answer: we must share our knowledge, concepts, and perspectives. Journals can do this best by publishing comprehensive up-to-date summaries from leading experts in the respective fields. Such conceptual reviews should be understandable by every scientist with basic knowledge in physiology, and readable in the limited time which we can devote to studies outside our specific areas of expertise. Ideally, it should be fun to read them.

With our new “Concept Article” series, we aim at meeting all these needs. Renowned scientists highlight the most important facts and findings in critical topics - the typical “neuralgic points” which we encounter during teaching or when searching for solid information outside our specific field of expertise. For example, a person working on the kidney may want to know the present concept of neocortical organization (“is there a canonical circuit, yes or no?”), or a neurophysiologist looks for an update on renin release (“what shall I tell my students about the underlying signaling pathways?”). We all encounter such questions, especially in fields which are rapidly moving. In most of the cases, we do not have the time to read an in-depth review or do a long search in Pubmed.

This is where “Concept Articles” come into play. They will provide short, comprehensive overviews of well-defined hot topics, give state-of-the-art answers to the most pressing questions, and provide well-designed illustrations which can be used for teaching. With this, they are more up-to-date than typical textbooks, but less demanding and time-consuming than reviews written for specialists. In any case, we want to address and fill the most critical gaps, some of which we have already identified in the Board of Editors. However, we certainly have biases and blind spots, so suggestions from our readers are welcome!

We hope you will enjoy reading Concept Articles, and feel that they support your teaching, writing, and, ideally, cooperating with fellow scientists. And, of course, we encourage you to suggest an own contribution to this series, to make your specific area of research visible and accessible to your colleagues!

